# Core concepts in statistics and research methods. Part 2: clinical research principles and observational studies

**DOI:** 10.1016/j.bjae.2024.11.006

**Published:** 2025-01-15

**Authors:** D.W. Hewson, B. Stuart

**Affiliations:** 1 Nottingham University Hospitals NHS Trust, Nottingham, UK; 2 University of Nottingham, Nottingham, UK; 3 Queen Mary University of London, London UK

**Keywords:** clinical study, observational study, regression analysis, research design

**Learning objectives**

By reading this article, you should be able to:

• Classify clinical research designs in anaesthesia and critical care commonly reported in the literature.
• Outline the ethical principles governing clinical research.
• Compare the comparative strengths and weaknesses of observational and experimental research methods.
• Describe the statistical approaches to minimise confounding and bias in observational data.
• Explain the purpose and broad content of relevant reporting standards for clinical research studies.

**Key points**

• Primary clinical research can be categorised as either observational or experimental.
• Bias is present in all observational studies.
• The relationship between an exposure and outcome can be distorted by a third extraneous variable, a confounder.
• Statistical strategies to address confounding include matching, stratification, regression, propensity scoring and instrumental variable analysis.
• The main weakness of propensity scoring is that it can only control for known and measured confounding.

Clinical research in anaesthesia, critical care and pain medicine, systematically studies people to understand health and disease relevant to our specialties. The common aim of all clinical research is to improve the way health and illness are managed for the benefit of the general population. Participants in clinical research are often patients, but can be healthy subjects or healthcare professionals themselves.

Although informal (‘trial and error’) tests of medicines and diets have been recorded as far back as the pre-Christian era, the Scottish physician James Lind is widely credited as the originator of the systematic clinical experiment after he divided a group of 12 scorbutic sailors to assess the impact of dietary intervention on scurvy in 1747.^1^ Further landmark clinical studies include those of Semmelweis on maternal sepsis (1861) and Snow on cholera (1855). Of note, the published results were widely ignored or rejected by contemporaries in all three historical cases.

This article, part of the *BJA Education* series ‘Core concepts in statistics and research methods’ provides an overview of clinical research designs, focusing on the statistical principles of observational research. An accompanying article on experimental clinical research designs (including the randomised trial) will follow.

## Clinical research design

Clinical scientific endeavour can be divided into primary research, which reports and interprets new data directly produced by undertaking the research; and secondary research, which gathers and analyses data previously reported, often by other researchers, to generate new interpretations. An example of secondary research is a systematic review, with or without meta-analysis. Secondary research methods and interpretation will be considered in a forthcoming article in this journal.

Clinical studies generating new data, that is to say primary research, can be categorised as observational or experimental. In an observational study, researchers do not assign participants to an exposure, intervention, or treatment as part of the research protocol, whereas in experimental research participants are allocated to one or more interventions by the research team. In both observational and experimental research we often wish to study a specific outcome (the ‘dependent variable’) and study its relationship to the exposure or treatment (the independent variable). One type of experimental study, commonly conducted in clinical research, is the randomised trial. The term ‘clinical trial’ is often used to mean research with an experimental, rather than observational, design. This nomenclature is used in this and a future article in this series on randomised trials.

**Fig 1:**
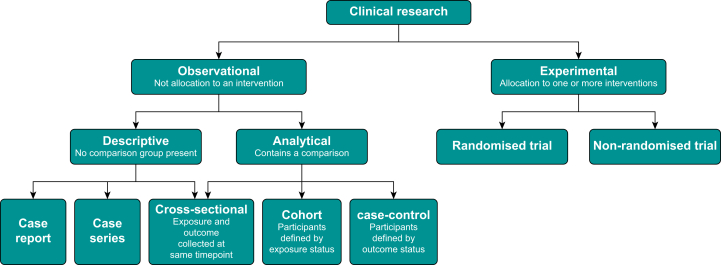
General taxonomy of primary clinical research.

Another way to classify clinical research is according to the intended clinical question. Much research is devoted to assessing the impact of treatment on ill health, but investigations of disease prevention, diagnostic testing and to clarify genetic or epidemiological uncertainty are also common. A general taxonomy of primary clinical research is shown in Figure 1. Importantly, data generated from research may be either quantitative (i.e. numerical) or qualitative (i.e. narrative). Many research studies collect both forms of data to report a mixed methods analysis, which draws on the strengths of each type of information.

### Ethical principles of clinical research

The ethical principles underpinning clinical practice, namely respect for individual autonomy, beneficence, non-maleficence and justice, are also fundamental to the design and conduct of good research and medical statistics. The principles of ethical human research were first outlined in the 1947 Nuremberg Code, subsequently organised in the 1964 Declaration of Helsinki (most recently updated in 2013) and implemented in European and American jurisdictions by Good Clinical Practice regulations.^2^, ^3^, ^4^ Although arrangements of ethical approval of clinical research vary by country, the principle that all research involving human participation or personal data (whether observational or experimental) requires careful consideration of ethical implications before it commences is universal. Such scrutiny should be both internal (i.e. ethical risks posed by the study considered by the researchers themselves) but also administered externally (e.g. by a specialised research ethics committee otherwise independent of the study and investigators). In the UK, ethical scrutiny of clinical research connected with National Health Service (NHS) patients is provided by a network of more than 80 multidisciplinary NHS Research Ethics Committees (RECs), organised by the NHS Health Research Authority and obligated to provide opinion within a statutory 60-day timeline. Ethical review of medical research falling outside the remit of NHS RECs (i.e. not connected in any way with NHS patients) is usually scrutinised by University or other institution-specific ethics committees. Poorly conducted studies, for example with imprecise aims or hypotheses, inappropriate designs, defective data management leading to data falsification, badly applied statistical methods etc, are themselves unethical because they waste resources and may lead to incorrect conclusions harmfully influencing clinical practice or policy. It is therefore an ethical imperative that research is conducted to a satisfactory standard.

## Observational study design

Observational studies intend to provide useful data on the characteristics of samples of participants from a population. By virtue of their lack of assignment of participants to an intervention, observational studies are inherently unable to draw conclusive causative inferences on treatment-outcome relationships. The volume and quality of routinely collected health data has increased in recent decades (in part because of electronic data storage), which invites observational analyses on available data seeking answers to important research questions.

Observational studies may have several advantages over experimental research, such as the randomised trial. Observational studies commonly describe much larger samples than randomised trials and so can usefully explore rare outcomes. Their non-interventional nature means they can be comparatively inexpensive and less time-consuming to conduct compared with randomised trials. Certain research questions may be fundamentally unethical to answer by an interventional study design, or involve variables (such as inherited traits) that are impossible to manipulate, and so observational research is sometimes the only reasonable option to advance science on certain topics.

A summary of common quantitative observational study designs in anaesthetic research is found in Table 1. Although it has been proposed that prospectively conducted cohort studies should be ranked highest in the hierarchy of observational evidence, followed by retrospective cohort studies, case-control studies, cross-sectional studies then case series, in practice it is more important to acknowledge that different designs may be best suited to answering different research questions, and to appraise the specific methodological and analytical strengths and weaknesses of any individual study before determining its place in the wider literature.^9^

**Table 1:** Common quantitative observational study designs in anaesthetic and critical care research with illustrative examples of each study type.

Study design	Purpose	Strengths	Weaknesses	Example drawn from the recent specialty literature
Case report or series	Description of one or more clinical vignettes from a real-world setting.	Useful to illustrate novel and unusual features in patients in the context of existing knowledge on a topic, which may warrant subsequent further study by other methodologies.	No control comparison, limited generalisability.	Alexeev M, and colleagues. *Carbon dioxide embolism during posterior retroperitoneal adrenalectomy.*^5^
Cross-sectional	A description of outcome, exposure data, or both from individuals collected at a single time point. A ‘snapshot’ of a population at a specific time.	Commonly deployed to determine prevalence. Describes routinely collected health data, research-specific data, or both as a population-based description of current practice.	Cannot determine causal relationships between exposure and outcome.	Walker EMK, and colleagues. *Patient reported outcome of adult perioperative anaesthesia in the United Kingdom: a cross-sectional observational study.*^6^
Cohort	Participants are included based on their exposure status (‘exposed’ or ‘unexposed’) and either retrospective or prospective longitudinal data described.	Can estimate the incidence of multiple outcomes and risk factors linked by temporality (i.e. participants at the time of enrolment had the exposure but not the outcome).	Follow-up of participants over time (longitudinally) can be expensive, time-consuming and incur data loss. Selection bias must be minimised by ensuring exposed and unexposed participants are similar in all important regards except for their exposure status.	Robba C, and colleagues. *Intracranial pressure monitoring in patients with acute brain injury in the intensive care unit (SYNAPSE-ICU): an international, prospective observational cohort study.*^7^
Case-control	Participants are defined by their outcome status, for example presence of a condition or disease (‘cases’) that has already occurred and compared with subjects without the outcome (‘controls’) to seek exposure risk factors.	Can suggest associative relationships but not establish causation. Retrospective collection of exposure data is usually less time-consuming than prospective study, especially for rare outcomes.	Choice of control group vitally important to prevent selection bias as controls must be similar to cases except for the outcome in question. Cannot provide incidence rates.	Rujirojindakul P, and colleagues. *Risk factors for reintubation in the post-anaesthetic care unit: a case–control study.*^8^

### Comparison of case-control and cohort studies

The most prevalent designs in observational clinical research are case-control and cohort studies, although these labels are frequently misapplied. The important difference between the two methodologies is that case-control studies enrol participants based on their *outcome* status (with or without a disease or outcome) and seek exposure risk factors, whereas a cohort design enrols on *exposure* status (without or without a risk factor) and describes one or more subsequent outcomes. As a consequence, rare outcomes are frequently examined via case-control design but, conversely, rare exposures are best examined by a cohort study. Both designs can be subject to prospective or retrospective data aggregation, although cohort studies are more frequently prospective and case-control studies more frequently retrospective. Whether a cohort study is designated ‘prospective’ or ‘retrospective’ is most straightforwardly determined by the manner in which exposure data are gathered in relation to the subject outcome. In a prospective cohort subjects are enrolled and exposure data collected before subjects develop the outcome of interest. In a retrospective cohort enrolment and exposure data are established after subjects have already developed the outcome. Sometimes studies labelled as ‘retrospective’ (e.g. research using previously collected large registries or repositories of health data) could in fact by designated ‘prospective’ in that such exposure data were collected from subjects before an outcome of interest occurred and before the research was conceived.

Case-control studies often report the proportion of subjects with (‘cases’) and without (‘controls’) an outcome who experienced one or more exposure variables. This relationship is best expressed as an odds ratio (OR) with 95% confidence interval (95% CI). Case-control studies cannot establish the prevalence or the incidence of outcomes. In contrast, a cohort study can report the incidence of an outcome in an exposed group and compare this with the outcome incidence in a non-exposed group, producing a relative risk (RR) with 95% CI. Means of calculating OR and RR have been detailed in a previous article in this series.^10^

### Association, correlation and causation

The terms ‘association’, ‘correlation’ and ‘causation’ have distinct meanings in clinical research.^5^^,^^11^ Association is the most general term and means only that one variable provides information about another variable. An associative relationship does not need to be linear. Correlation describes an increasing or decreasing linear trend between variables and thus implies association. Correlation between linearly related, normally distributed, variables can be quantified, in terms of direction and strength, by a Pearson correlation coefficient. Non-normally distributed data are better assessed by the Spearman rank correlation coefficient, which requires no assumptions of normality. Correlation coefficients, which are calculated from −1 (perfect negative correlation) to +1 (perfect positive correlation), should always be reported with a 95% CI to provide bounds of certainty in the reported results. Correlation does not predict how one variable causes another one to change and correlation is not causation.^12^ Causation occurs if one variable *relies on* another variable for its value.^13^ Causation requires an association between variables but does not imply correlation. Using obstructive sleep apnoea (OSA) as an example, we can say OSA is *associated* with age because there is a non-linear (bimodal) relationship between presence of OSA and age in years. We may also state that OSA severity is *correlated* with body mass index, and that OSA *causes* postoperative pulmonary complications.

Causality is usually assessed by the experimental randomised trial. However, causal inference can be speculated from observational data using a variety of possible criteria and statistical techniques reviewed elsewhere.^14^, ^15^, ^16^ Such causal speculation from observational evidence may be subsequently sustained or overruled by later experimental studies.

### Bias in observational studies

Some degree of bias is present in all observational studies. Bias can be defined as systematic error or deviation leading to a distortion of the true effect of any given exposure factor on a specific outcome.^17^ Bias undermines the internal validity of observational research by impeding the reader's confidence that the study has measured what it aimed to measure.^18^ Bias can also affect external validity if the reported results cannot be usefully generalised from the studied sample to the wider population. Biases may increase or decrease the sample data RR or OR reported in studies, the direction of change depending on the specific bias under examination and this direction may not be predictable in advance. It is not unusual for observational analyses examining identical clinical questions to produce discordant results. Discordant results may be because of how each analysis has mitigated potential biases. An example from the recent anaesthesia literature is the comparative benefit of sugammadex *vs* neostigmine for the prevention of postoperative pulmonary complications. Two large observational datasets have produced differing conclusions and these differences could plausibly be ascribed in part to their respective approaches to handling confounding variables and reducing bias.^19^, ^20^, ^21^ It is critical that researchers consider and seek to minimise as much as possible the different types of bias set out below.

#### Selection/attrition bias

In broadest terms, selection bias occurs when a study sample does not accurately represent the target population.^22^ Truly random study sampling from a population of interest is usually not possible in clinical observational research, as it is more common to execute selected sampling using data that has already been collected from the population. In cohort and case-control studies, selection bias most seriously compromises internal validity when the sampling technique results in groups being different in one or more important regards other than their exposure or outcome status, respectively. For example, in a hypothetical prospective cohort study of quality of recovery (outcome) after surgery and general anaesthesia (exposure) requiring the administration of postoperative written questionnaires, patients with high literacy and previous educational attainment may be more likely to submit a complete dataset, resulting in potential bias. Selection bias can also limit external validity, for example when the sample is derived from a single institution making generalisability to other healthcare settings challenging.

#### Measurement bias

Measurement bias describes systematic errors in the measurement or classification of either exposures or outcomes and is also known as information or observation bias. In cohort and case-control studies, measurement bias is minimised by collecting information from all participants, regardless of group, in the same manner. For example, in a hypothetical cohort study of accidental awareness during general anaesthesia (AAGA; outcome) after elective general surgery (exposure), patients undergoing day-case procedures may report lower rates of AAGA when answering a remote written assessment, compared with patients who underwent inpatient surgery and subsequently undergo in-person follow-up interview with a healthcare professional. The method of determining whether the outcome has occurred may itself induce error in the results.

#### Confounders, mediators, moderators and colliders

Most observational studies attempt to define the strength of relationship between an exposure and an outcome. A confounding variable affects the outcome in question and is related in some non-causative manner to the measured exposure variables.^23^ The relationship between exposure and outcome is thus distorted by the effect of the third extraneous variable—the confounder. This effect is particularly troublesome when the confounding variable is present to a varying extent in different study groups. A key strength of randomised clinical trials over observational studies is that randomisation itself prevents non-random unequal distribution of confounding variables between groups and therefore eliminates confounding as a methodological concern. Confounding can thus be considered the central methodological difficulty of observational studies. In a hypothetical observational examination of renal complications (outcome) after antibiotic prescription (exposure) in critically ill patients, illness severity can be anticipated as a confounding variable, because the most unwell patients are likely to receive more antibiotics and are also expected to sustain a higher rate of complications. The study must therefore control for illness severity to delineate the underlying relationship between exposure and outcome.

Unlike a confounder, a collider variable is one which itself causally arises from both the exposure and outcome. If such a collider is statistically controlled for (in the manner of a confounder) a distorted association between exposure and outcome may arise. Misidentification of a collider variable as a confounder with subsequent controlling according to the methods outlined below itself induces bias.

**Fig 2:**
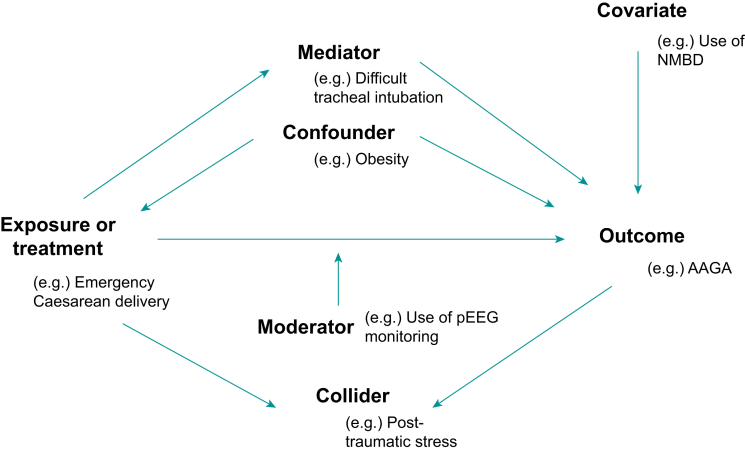
Simplified causal diagram illustrating confounding, mediating and moderating variables affecting the relationship between exposure and outcome, with a theoretical example from anaesthesia and critical care. AAGA, accidental awareness under general anaesthesia; NMBD, neuromuscular blocking drug; pEEG, processed electroencephalography.

In further contrast to a confounder, which exerts an effect on both exposure and outcome, a mediator is a variable that intervenes between exposure and outcome. A mediator arises from the exposure and precedes the outcome. Another type of variable affecting the exposure–outcome relationship is a moderator. A moderator variable influences the direction or strength of the relationship between exposure and outcome. Figure 2 illustrates these concepts in a basic causal schematic with putative examples of each variable in the context of an observational study of accidental awareness under general anaesthesia in parturients.

An extreme example of the harmful effect of confounding on data interpretation is Simpson's paradox. Simpson's paradox describes the phenomenon of a relationship between exposure and outcome variables being entirely reversed depending on whether analysis is conducted at an aggregated or a stratified level. Box 1 provides a hypothetical illustrative example of this phenomenon.

Box 1 **The harmful effect of confounding—a worked example of Simpson's paradox**

Consider a hypothetical prospective cohort study comparing thoracic epidural analgesia (TEA) with paravertebral blockade (PVB) to prevent severe pain on coughing (11-point numeric rating score ≥7) in 1000 adults after blunt force chest wall trauma. Five hundred patients received TEA and 500 patients received PVB.

The following contingency table summarises the success rates of these analgesic techniques, expressed as number (%) of patients experiencing severe pain 24 h after injury, stratified by number of rib fractures sustained. TEA is associated with higher success rates (i.e. reduced occurrence of severe pain) for both lower (6.9% *vs* 12.8%; relative risk [RR]=0.54) and higher (27.3% *vs* 32.8%; RR=0.83) numbers of rib fractures, but TEA appears less effective than PVB if number of fractured ribs is not considered (21.4% *vs* 15.4%; RR=1.39).

This paradox arises because the probability of a patient receiving either TEA or PVB depended on the number of fractured ribs sustained and this confounding variable was disproportionately present between treatment groups. Most patients with ≤4 fractured ribs (i.e. 436/581, 75%) received PVB whereas sustaining >4 rib fractures more commonly (355/419 or 84.7%) led to TEA insertion.

**Table:** 

	TEA	PVB	
Number of fractured ribs ≤4	10/145 (6.9)	56/436 (12.8)	RR = 0.54
Number of fractured ribs >4	97/355 (27.3)	21/64 (32.8)	RR = 0.83
Any number of fractures ribs	107/500 (21.4)	77/500 (15.4)	RR = 1.39

Alt-text: Box 1

### Methods to control for confounding in observational studies

Measures to reduce selection and measurement bias must be instituted in the design phase of an observational study. Similarly, possible confounding factors should be anticipated during study design to allow collection of these variables and subsequent control by a variety of possible statistical adjustments. Unknown or unmeasured confounders cannot be adjusted for, so careful consideration of possible confounders at the study planning phase is important. Adjustments usually aim to isolate the effect of one or more exposures on the outcome of interest by holding other, potentially confounding factors, constant during analysis. In increasing order of complexity, we present a summary of common adjustment techniques in observational research below.

#### Matching

Matching can be applied prospectively or retrospectively to cohort or case-control studies by identifying key confounders and ensuring participants enrolled to study groups are matched by these characteristics. Matching can be conducted on an individual (also known as a ‘one-to-one’ or ‘pairwise’) or frequency basis. Individual matching pairs each study subject with a comparator subject sharing the matched characteristic(s). Frequency matching intends to ensure the frequency of nominated confounders is equal between groups. For example, in a case-control study of patients' age and perioperative myocardial injury, individual matching might pair participants undergoing the same type of operation, preoperative smoking and diabetes mellitus status (all potential confounders). In contrast, frequency matching aims for these confounders to be present in equal proportions overall in both ‘case’ and ‘control’ groups. If conducted correctly, matching will result in no statistical difference in the prespecified confounder between study groups. The main limitations of matching are practical and analytical. From a practical perspective, matching increases the difficulty of recruitment into the study, especially if multiple matching characteristics are proposed, as each participant needs to share characteristics with their opposing matched subject. Matching difficulty can be mitigated by applying a degree of reasonable flexibility in the matched characteristic; for example, accepting a matched body mass index range within plus or minus 5 kg m^−2^, rather than a single value. But nevertheless, matching can only be conducted on a limited number of potential confounders, sometimes far fewer than could reasonably be supposed to exist in a given clinical scenario. The analytical limitation imposed by matching is that the matched characteristics themselves cannot be examined with regard to their effect on outcome (in a cohort) or exposure (in a case-control study).^18^

#### Stratification

Analysis by stratification involves grouping sample data by potential confounding variables (into ‘strata’) and undertaking analysis by these subgroups to seek the relationship between exposure and outcome unmodified by that confounder. The main limitation of stratification is that while it is straightforward to undertake and interpret in the presence of only one or two potential confounders, it becomes unwieldy if many potential confounders are reported, especially if each confounder itself necessitates reporting of multiple relevant levels (e.g. body mass index grouped by <18.5, 18.5–24.9, 25–29.9, 30–34.9, 35–39.9, >40 kg m^−2^), in contrast to simple dichotomised data (e.g. biological sex: male, female). For example, stratification adjustment for a hypothetical observational study examining the relationship between surgical site infection (outcome) and presence of diabetes mellitus (exposure) where biological sex, body mass index and smoking status (current, former or never) were offered as potential confounders, would require the reporting of 36 substrata. The more substrata analyses are undertaken, the smaller the corresponding sample size and the increased risk that a positive finding may arise purely be chance. To control simultaneously for multiple confounders, regression analysis is frequently used.

#### Regression analysis

In observational research, regression analysis is commonly used to model the relationship between an outcome (dependent variable) and one or more exposures (independent variables). Such techniques are useful to control for confounders, but also to make predictions based on observed data. The relationship between a single continuous, dichotomous or categorical exposure variable and a continuous outcome can be quantified by linear regression producing a regression coefficient (β) indicating the direction and strength of the relationship, and associated 95% CI. At its most basic, linear regression represents the plotting of a line of best fit though two variables graphically represented on a scatter plot and will allow an estimate of how much the outcome variable changes for every unit change in exposure variable. In the circumstance of a dichotomous outcome variable (e.g. dead or alive at a specific time point), logistic regression analysis can be used. Logistic regression provides the relative probability (OR) of experiencing the outcome for different levels of the exposure.

Linear and logistic regression can be extended to explore the relationship of multiple simultaneous exposure variables on the outcome of interest, known as multivariable regression analysis. Multivariable regression estimates the contribution of each exposure variable while controlling for the other independent variables (including possible confounders) by holding them constant. The ability of each type of regression analysis to accurately model data relies on certain assumptions specific to the model about the observed sample data. For example, in multivariable linear regression these are that the regression coefficients are linearly related, that all differences between observed and modelled values are normally distributed with a fixed variance (normality and homoscedasticity) and that sample observations are independent from one another (independence).^24^

The sample size must be considered when planning a multivariable regression model. Broadly, the more independent variables that are included in a regression analysis, the larger the sample size that may be required. For example, it is usually not appropriate to test for 10 variables in a regression model (e.g. age, sex, weight, height, ASA score, duration of anaesthesia, presence of neuraxial analgesia, intraoperative opioid use, surgical complexity, length of inpatient stay) in a dataset of only 50 subjects. Statistical input should always be sought to ensure an analysis is adequately powered. Transparent reporting of regression analysis should always confirm the assumptions upon which it is conducted (which can largely be done by examination of a scatter plot) and model goodness-of-fit (the detailed description of which is beyond the scope of this article). Failure to do so may increase uncertainty associated with the regression data.^24^ A fuller exposition of regression analysis will feature in a forthcoming article in this journal.

#### Propensity scoring

**Fig 3:**
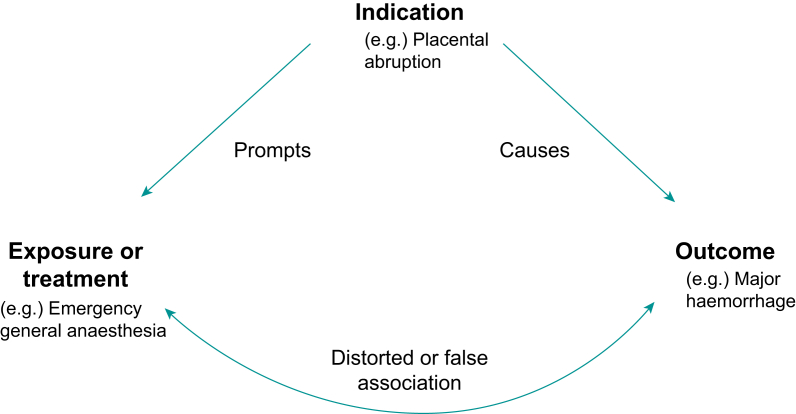
Confounding by indication, with an example from anaesthesia and critical care.

Propensity scoring extends matching, stratification and regression, as described above, by summarising multiple measured confounders into a single value.^25^ This value, the propensity score, is the probability (defined from 0–1) a subject receives a given treatment conditional on measured baseline characteristics including identified confounders.^26^ The purpose of propensity matching is to limit confounding by indication, a concept illustrated in Figure 3.^27^ The decision of which variables to include in a propensity score model can be facilitated by construction of causal directed acyclic graphs, which serve to collate relationships between exposure, outcome and other variables of interest, including (among others) confounders, mediators and moderators.^28^ The propensity score is usually calculated using logistic regression with treatment/exposure as the dependent variable and the baseline characteristics and specified confounders which predict the treatment/exposure as independent variables. The effect of a treatment intervention is then estimated among subjects with the same propensity score, thereby controlling the bias induced by those confounding variables. One advantage of propensity scoring over multivariable regression is its ability to account for more potential confounders than can typically be accommodated using regression analysis in circumstances when the outcome is rare. Another advantage of propensity scoring is that, unlike multivariable regression, propensity scoring is separate from outcome analysis and therefore less likely to be influenced by a researcher's expectations and bias.

Matching subjects by propensity score creates balance between groups for confounders, and therefore between-group differences in outcome can be ascribed with less bias to direct treatment effect.^29^ The disadvantage of propensity score matching is the necessary exclusion of non-matched subjects, leading to loss of sample size, power and precision of estimates. Use of matching for propensity score analysis has been criticised as unintentionally increasing bias, to the extent that some authors recommend that matching is not used as part of a propensity score analysis.^30^

Multiple alternative techniques exist to handle propensity scores that do not rely on subject matching, including propensity score stratification, weighting and covariate adjustment. The execution and comparative advantages and disadvantages of these methods are beyond the scope of this article but have been reviewed in detail elsewhere.^31^^,^^32^ The method used to apply propensity scoring can markedly affect the arising study results and so expert statistical input should be sought for all planned propensity analyses.^33^, ^34^, ^35^, ^36^

Propensity scoring imitates the inherent facility of randomised trials to balance confounders between groups.^37^ The chief difference, and therefore weakness, of propensity scoring is that whereas randomisation controls for both measured and unmeasured confounders, propensity scoring can only control known and measured confounding variables. When presented with propensity scored analyses it is important to assess the balance of baseline characteristic variables between study groups. Balance may be assessed most straightforwardly by comparing summary statistics such as mean values or proportions. Many analyses present standardised differences (i.e. for any given variable the difference between groups divided by the pooled standard deviation of both groups) and a difference of <0.1 is usually considered unimportant.^38^ It is also vital to consider which variables have not been included in the propensity score (either by deliberate exclusion by the researchers, or because they were unmeasured), as unaccounted confounding variables will exert a biasing effect on study results.

#### Instrumental variable analysis

Given the fundamental weakness of propensity scoring—that it can only attempt to account for known and measured confounding factors—a statistical technique capable of controlling both known and unknown variables would offer a substantial benefit. Instrumental variable analysis seeks to achieve this by identifying a variable (the ‘instrumental variable’) which is associated with the treatment/exposure but has no direct association with the outcome, except through its influence on treatment. A recently published example in the anaesthetic literature sought to examine the relationship between intraoperative hydromorphone administration dose (exposure) and postoperative pain (outcome) in the presence of multiple possible unmeasured confounders.^39^ The unit dose of hydromorphone contained in single vials and available to clinicians was shown to be associated with subsequent dose of drug given during surgery (exposure) but it is not, in itself, related to pain scores (outcome). Unit dose of hydromorphone could therefore serve as the instrumental variable, facilitating an estimation of the exposure–outcome relationship without confounding. The main weakness of independent variable analysis is that identifying a suitable independent variable, fulfilling the various assumptions required, is often difficult. Detailed review of this technique is available to interested readers.^40^

## Reporting and interpretation of observational studies

By providing a standardised structure to manuscripts intended for publication, reporting guidelines improves understanding and appraisal of clinical observational research. A major barrier to evidence synthesis and generation of high-quality meta-analysis is incomplete or inadequate reporting in primary literature. The ‘Enhancing the quality and transparency of health research (EQUATOR) network’ provides a database of >200 reporting guidelines for observational research, *primus inter pares* the ‘Strengthening the reporting of observational studies in epidemiology (STROBE) statement’.^41^ STROBE, and its subsequent extensions, can be applied to a wide variety of observational study designs, and are usually mandatory components of manuscript submission for peer review to high impact specialty journals. Specific statistical techniques, such as propensity scoring, should be reported to published standards, where these exist, and should be sufficiently detailed to allow replication of the analysis by readers.^42^^,^^43^

Interpretation of observational research findings should always pay regard to associative, rather than causative, nature of the observed exposure–outcome relationship. When evaluating an item of observation research, we believe clinicians should pay regard to the following fundamental aspects of any study: the clarity of the research question, aims or objectives and whether the chosen study design is capable of providing insight into these stated uncertainties; whether justification for the sample size is provided and important potential biases are addressed in the design analyses; the consistency and simplicity of communication of the study results with careful acknowledgement of data limitations and contradictions.

## Conclusions

The complexities of observational research should not be underestimated: the above description of biases and their means of control are far from comprehensive. Planning, conducting and reporting such research requires clinical subject matter experts to work closely with statisticians so that appropriately careful conclusions are drawn about the relationship between studied exposure and outcome.

## MCQs

The associated MCQs (to support CME/CPD activity) will be accessible at www.bjaed.org/cme/home by subscribers to *BJA Education*.

## Declaration of interests

DWH is an editor and editorial board member of *BJA Education*. BS declares no relevant conflicts of interest.
